# Methylene blue induced morphological deformations in *Plasmodium falciparum* gametocytes: implications for transmission-blocking

**DOI:** 10.1186/s12936-017-2153-9

**Published:** 2018-01-08

**Authors:** Ishan Wadi, C. Radhakrishna Pillai, Anupkumar R. Anvikar, Abhinav Sinha, Mahendra Nath, Neena Valecha

**Affiliations:** 1 Indian Council of Medical Research-National Institute of Malaria Research, Sector 8, Dwarka, New Delhi 110077 India; 2 Department of Chemistry, University of Delhi, Delhi, 110007 India

**Keywords:** Malaria, Gametocytes, Morphological deformations, Microscopy, *P. falciparum*, Gametocytocidal activity, Methylene blue, Primaquine, G6PD deficiency

## Abstract

**Background:**

Malaria remains a global health problem despite availability of effective tools. For malaria elimination, drugs targeting sexual stages of *Plasmodium falciparum* need to be incorporated in treatment regimen along with schizonticidal drugs to interrupt transmission. Primaquine is recommended as a transmission blocking drug for its effect on mature gametocytes but is not extensively utilized because of associated safety concerns among glucose-6-phosphate dehydrogenase (G6PD) deficient patients. In present work, methylene blue, which is proposed as an alternative to primaquine is investigated for its gametocytocidal activity amongst Indian field isolates. An effort has been made to establish Indian field isolates of *P. falciparum* as in vitro model for gametocytocidal drugs screening.

**Methods:**

*Plasmodium falciparum* isolates were adapted to in vitro culture and induced to gametocyte production by hypoxanthine and culture was enriched for gametocyte stages using *N*-acetyl-glucosamine. Gametocytes were incubated with methylene blue for 48 h and stage specific gametocytocidal activity was evaluated by microscopic examination.

**Results:**

*Plasmodium falciparum* field isolates RKL-9 and JDP-8 were able to reproducibly produce gametocytes in high yield and were used to screen gametocytocidal drugs. Methylene blue was found to target gametocytes in a concentration dependent manner by either completely eliminating gametocytes or rendering them morphologically deformed with mean IC_50_ (early stages) as 424.1 nM and mean IC_50_ (late stages) as 106.4 nM. These morphologically altered gametocytes appeared highly degenerated having shrinkage, distortions and membrane deformations.

**Conclusions:**

Field isolates that produce gametocytes in high yield in vitro can be identified and used to screen gametocytocidal drugs. These isolates should be used for validation of gametocytocidal hits obtained previously by using lab adapted reference strains. Methylene blue was found to target gametocytes produced from Indian field isolates and is proposed to be used as a gametocytocidal adjunct with artemisinin-based combination therapy. Further exploration of methylene blue in clinical studies amongst Indian population, including G6PD deficient patients, is recommended.

## Background

Malaria is one of the deadliest parasitic diseases on the planet imposing a heavy socio-economic burden in developing countries, particularly in sub-Saharan Africa and South-East Asia. To achieve the dream of malaria elimination, preventing transmission is crucial and therefore approaches targeting gametocytes are highly essential. Transmission-blocking drugs that can effectively target mature gametocytes are very few, out of which only primaquine is licensed for clinical use [1]. Moreover, associated safety risks in glucose-6-phosphate dehydrogenase (G6PD) deficient patients have limited its usage on a large scale [2]. Although, in India, exact prevalence of G6PD deficiency is unclear but its overall magnitude is estimated to be 8.5% [3] which may rise even up to 27% in tribal population [4]. Further studies are urgently required to address safety and efficacy issues associated with primaquine [5]. In the meantime, research efforts should be directed towards developing new transmission-blocking drugs that are also safe for G6PD deficient patients.

Considering the cost, efforts and time involved in developing new therapeutic agents and bringing them to the market as safe and effective gametocytocidal drugs, a suitable alternative would be finding transmission blocking agents from the existing armamentarium. One such promising alternative is using an inexpensive but registered drug, methylene blue. Toxicity of methylene blue is dose and intrinsic G6PD activity dependent; similar to primaquine but with a difference that methylene blue exerts its oxidizing (haemolytic) properties at higher doses [6, 7]. In vitro and ex vivo studies performed with methylene blue against laboratory reference strains reported its late stage gametocytocidal activity [8–13]. Although a handful of reference strains such as 3D7 are useful to standardize and validate high throughput assays, a more realistic representation of drug efficacy is derived when potency of drug is evaluated on parasites that have been selected after years of multiple drug pressure and natural transmission. Evaluation of drug efficacy against gametocytes produced from these field isolates is of utmost importance as anti-malarial drugs respond differently to them as compared to culture adapted reference strains [13].

Here, in this study, a simple gametocyte production protocol for gametocyte producing field isolates is described. No complex downstream enrichment or purification steps are required and this protocol is also applicable to less affluent laboratory setups having minimum laboratory equipment. This is a pioneering study which establishes Indian field isolates as a model for gametocytocidal drug screening and evaluates gametocytocidal activity of a potential transmission blocking agent, methylene blue.

## Methods

### Cultivation of asexual stages and production of gametocytes

Asexual blood stages from infected blood collected from patients living in malaria prone areas of India—Rourkela (Odisha), Jaisalmer (Rajasthan), Jagdalpur (Chhattisgarh) and Mangalore (Karnataka), were cultivated in vitro by following the procedures of Trager and Jensen [14, 15] with minor modifications. The parasites were cultivated in RPMI 1640 medium (with glutamine) containing 25 mM HEPES, 2 g/L d-glucose, 2 g/L sodium bicarbonate, 40 μg/mL gentamicin sulfate supplemented with 10% heat inactivated AB^+^ human serum. A^+^ human blood at 10% haematocrit was used as a source of host erythrocytes. Cultures were maintained at 37 °C in the presence of 5% CO_2_. All culture adapted isolates were uniformly subjected to gametocyte production by following procedures of Ifediba and Vanderberg [16] with certain modifications. Parasites were maintained in culture starting from 0.5% parasitaemia (ring stage; sorbitol synchronized) and 10% haematocrit (day 0). These parasites were kept devoid of fresh erythrocytes throughout the course of 2 weeks of culture maintenance and daily replenished with complete RPMI-1640 media supplemented with hypoxanthine (50 µg/mL). Hypoxanthine provided additional purine source required for sexual differentiation as well as maturation of gametocytes. Haematocrit was reduced to 5% on day 8 and 50 mM *N*-acetyl-glucosamine (Sigma) was added on days 9–12 to eliminate asexual stage parasites. On day 14 onwards, a uniform population of gametocytes is obtained with majority of late stage (stage IV and V) gametocytes. Field isolates demonstrating higher gametocytaemia than the rest were classified as gametocyte producers and were selected for in vitro drug sensitivity testing. In separate set of experiments, these isolates were cultured continuously for a period of ~ 6 months from the date of revival of cryopreserved stabilate to ascertain the effect of number of in vitro asexual cycles on gametocyte production. Stability of gametocyte production phenotype across multiple cryopreservation events was also investigated. Comparative analysis was carried out using unpaired t-test and p value < 0.05 was considered as statistically significant. Drug susceptibility (asexual stages) of selected isolates to chloroquine and artesunate was also ascertained by microscopy-based schizont maturation inhibition assay [17].

### Gametocytocidal assays and data analysis

Stock solutions of methylene blue (Sigma-Aldrich) and primaquine (Sigma-Aldrich) were prepared in double distilled water and RPMI-1640 media, respectively. Appropriate working solutions were made afresh on the day of the experiment with complete culture medium. Gametocytes were harvested on the day of experiment and thorough and systematic morphological examination was performed by microscopy before carrying out screening experiments. Pre-dosed culture plates were prepared by plating two fold dilutions of the drugs in duplicates to achieve the desired concentrations up to 5 µM for methylene blue and up to 25 µM for primaquine and incubated with blood containing 2–3% gametocytes. Control wells were also prepared containing drug free media along with gametocytes for calculation of untreated inhibition. Also, 0.5% DMSO and 50 µM thiostrepton (Sigma Aldrich) were used as negative and positive controls, respectively. Plates were incubated at 37 °C for 48 h in presence of 5% CO_2_ [18]. After incubation period, thin smears were prepared, stained with 10% Giemsa and examined under a 100× oil immersion objective [19, 20]. Five thousand RBCs from each slide were counted to examine the gametocytaemia and gametocyte morphology at each concentration. Gametocytes observed were morphologically categorized into two groups, (1) normal morphology (NM) or (2) altered morphology (AM) and grouped either in early stage gametocytes (stages II and III) or late stage gametocytes (stages IV and V). Gametocytaemia for each concentration was expressed as percentage inhibition compared to drug-free control which was plotted against logarithm of drug concentration using a non-linear regression analysis (four parameter log dose with variable slope) to compute IC_50_ values and 95% confidence intervals. Dose–response curves expressed as percentage inhibition vs. logarithm of drug concentration were generated by Graphpad prism 6 [21]. IC_50_ values were calculated separately for early (stage II and III) and late (IV and V) stage gametocytes and in two categories. In the first category, only gametocytes bearing normal morphology were included and the 50% inhibitory concentration was labelled as IC_50_ (NM). In the second category, gametocytes with both normal and altered morphology were included and IC_50_ is designated as IC_50_ (Total) [10].

## Results

### In vitro gametocyte production

In present study, different field isolates demonstrated varied gametocyte production. Two isolates, RKL-9 and JDP-8 collected from Rourkela and Jagdalpur, respectively, exhibited higher gametocytaemia (> 2%) (Table 1) than other isolates in vitro and were therefore, deemed most suitable for stage specific drug screening experiments. Gametocytes produced from isolates RKL-9 and JDP-8 followed a very reproducible progression of gametocyte maturation from stage I to stage V. This was achieved in a period of ~ 12–14 days from the date of initiation of gametocyte culture (induction) and yielded > 70% of mature gametocytes. Both RKL-9 and JDP-8 were able to produce gametocytes after continuous maintenance in asexual culture for about 6 months as evidenced by statistically insignificant change (p > 0.05) in percentage gametocytaemia (Table 2). Gametocyte induction was not performed for the respective parasite lines beyond 6 months of continuous asexual culture due to technical reasons. Moreover, gametocyte production in these field isolates was found to be stable after multiple cryopreservation cycles. RKL-9 and JDP-8 remained high gametocyte producers even after three additional freeze–thaw cycles following initial cryopreservation of infected blood sample (time spent in asexual culture was up to 1 month before each cryopreservation event) (Table 2). Also, RKL-9 and JDP-8 were found to be chloroquine resistant with asexual stage mean IC_50_ value 174.7 nM. Artesunate was also tested against both of these isolates and was found to effectively inhibit the parasite growth with asexual stage mean IC_50_ of 4.32 nM. Individual asexual stage IC_50_ values of field isolates RKL-9 and JDP-8 are mentioned in Table 1. The rest of the field isolates used in this study exhibited comparatively lesser gametocytaemia (< 0.5%) in vitro.

**Table 1:** Percentage of gametocytaemia in Indian field isolates and their asexual stage drug susceptibility profile

Isolate	Place of origin	State	% Gametocytaemia	Chloroquine asexual stage IC_50_ (nM) (95% CI)	Artesunate asexual stage IC_50_ (nM) (95% CI)
RKL-9	Rourkela	Odisha	Total G: (2.50 ± 0.27)% Early G: (0.58 ± 0.12)% Late G: (1.92 ± 0.35)%	114.4 (77.70–168.4)	3.7 (2.710–5.036)
JDP-8	Jagdalpur	Chhattisgarh	Total G: (2.18 ± 0.23)% Early G: (0.50 ± 0.17)% Late G: (1.68 ± 0.24)%	266.7 (179.5–396.2)	5.06 (4.107–6.229)

Two field isolates collected from Rourkela and Jagdalpur demonstrated highest total gametocytaemia and were deemed suitable for drug screening experiments. Total G: % of gametocytes out of total erythrocytes represented as Mean ± SD, as a result of 6 separate induction experiments. Out of total gametocytes, percentage of stage I–III categorized as Early G (Mean ± SD) and percentage of stage IV, V categorized as Late G (Mean ± SD). Percentage gametocytaemia calculations are per 5000 RBCs

**Table 2:** Percentage of gametocytes produced from RKL-9 and JDP-8

Isolate	^A^Duration of asexual culture before induction (in months)		^B^Number of cryopreservation events before induction	
	0.5	6.5	1	4
RKL-9	2.24 ± 0.21	2.15 ± 0.42	2.40 ± 0.19	2.58 ± 0.46
JDP-8	2.02 ± 0.22	2.21 ± 0.23	2.28 ± 0.56	2.00 ± 0.39

Comparison of % gametocytaemia. ^A^At the interval of 6 months (expressed as mean ± SD of three separate induction experiments). ^B^After three cryopreservation events (expressed as mean ± SD of four separate induction experiments). No significant difference (p > 0.05) in percentage gametocytaemia after induction was observed in RKL-9 and JDP-8 for both the experiments ^A^(effect of duration of parasites in asexual culture) and ^B^(effect of number of cryopreservation events). Percentage gametocytaemia calculations are per 5000 RBCs

**Fig. 1:**
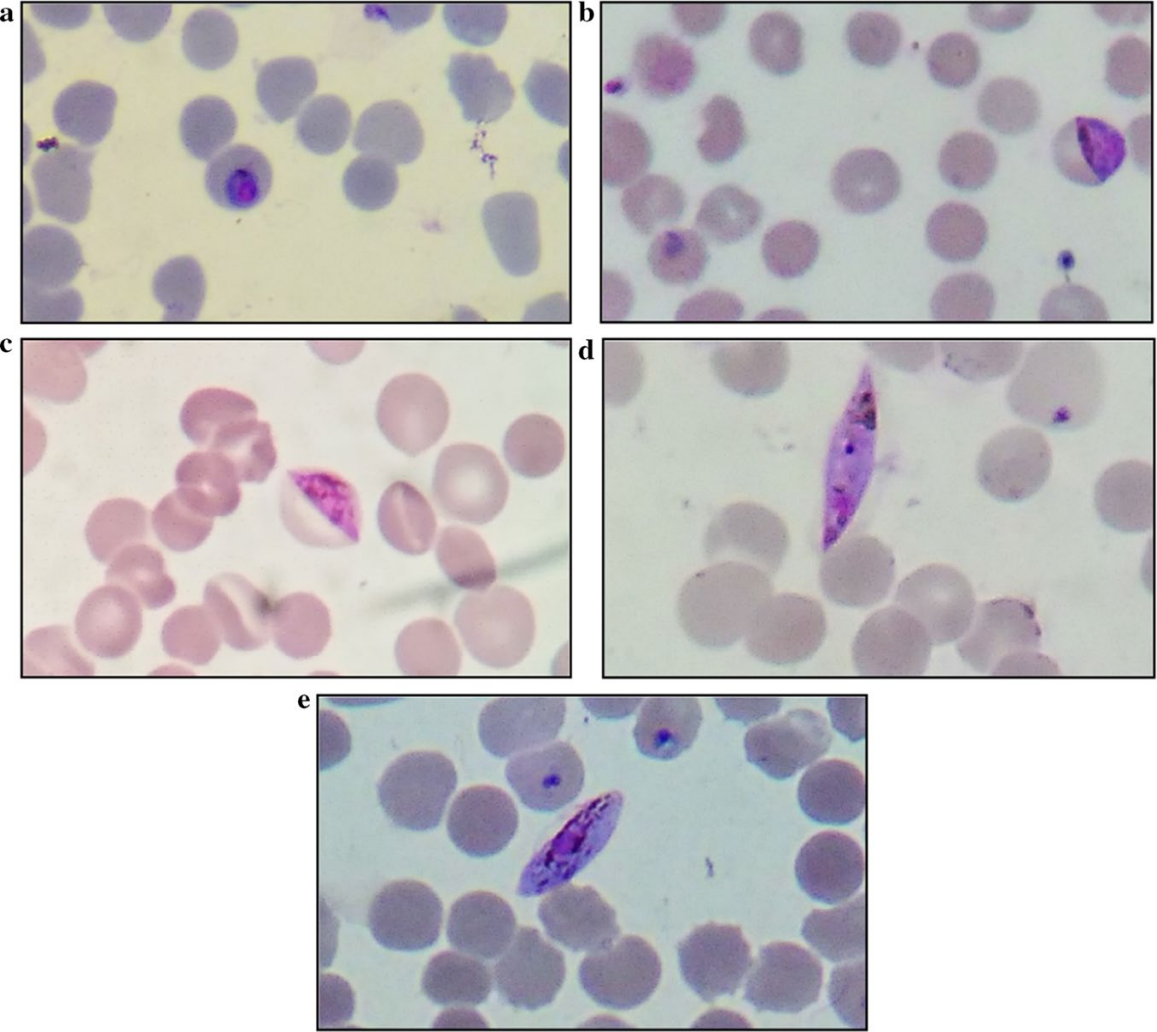
Representative images of different stages of gametocytes as observed under 100× objective bright-field light microscope. **a**–**e** Stage I–V gametocytes respectively (computer magnified image for better interpretation of morphology)

For evaluation of stage specific gametocytocidal activity of drugs discussed in subsequent section, gametocyte stages were categorized using a classification similar to one used by Carter and Miller [22] as seen from representative microscopic images shown in Fig. 1.

### Gametocytocidal assays using methylene blue and primaquine

**Fig. 2:**
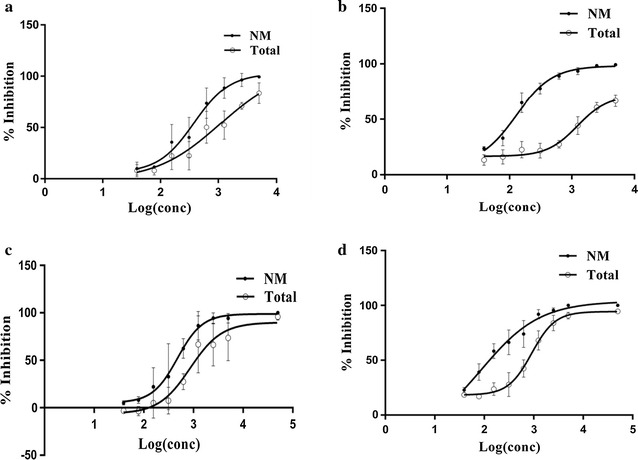
Dose-response curves describing relationship between concentration of methylene blue (in logarithmic scale) and percentage inhibition of **a** early stage gametocytes of RKL-9 **b** late stage gametocytes of RKL-9 **c** early stage gametocytes of JDP-8 **d** late stage gametocytes of JDP-8 (Error bars denote SD of the mean of three independent experiments). Gametocytaemia at start of drug screening experiments [In (Mean ± SD)%, RKL-9: Total G: (2.65 ± 0.36)%, Early G: (0.79 ± 0.14)%, Late G: (1.86 ± 0.23)% and JDP-8: Total G: (2.57 ± 0.35)%, Early G: (0.61 ± 0.18)%, Late G: (1.95 ± 0.21)%] where Early G and Late G represents the percentage of early (Stage II and III) and late stage (stage IV and V) gametocytes respectively and Total G represents total gametocytes inclusive of early and late stages. % Gametocytaemia calculations are per 5000 RBCs

**Fig. 3:**
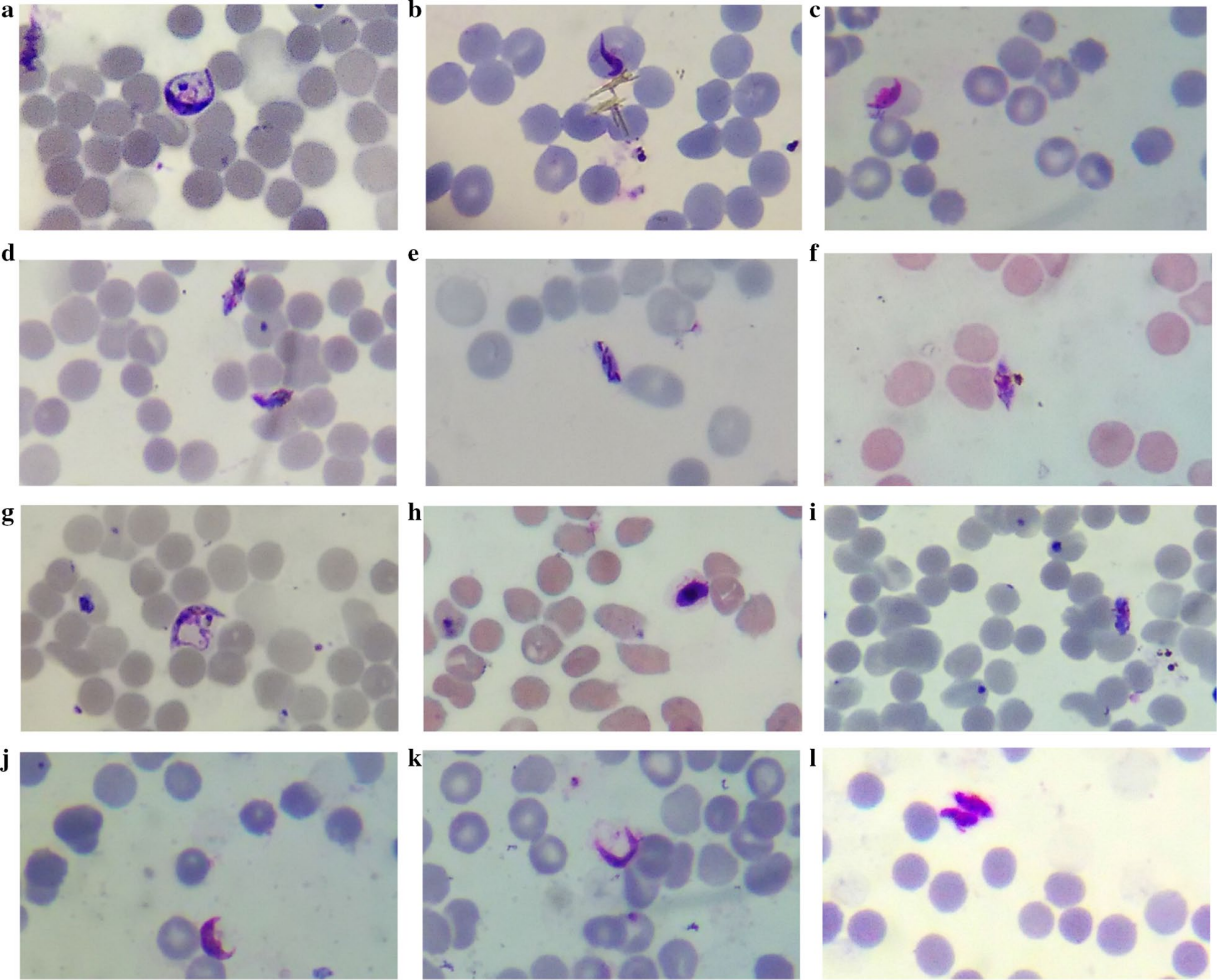
Microscopic images demonstrating morphological deformations in methylene blue treated early gametocytes (**a**–**c**) and late gametocytes (**d**–**l**). Deformations observed were in the form of (1) Shrinkage (2) Distortions (3) Membrane deformations, clearly representing unhealthy gametocytes (computer magnified image for better interpretation of morphology)

**Fig. 4:**
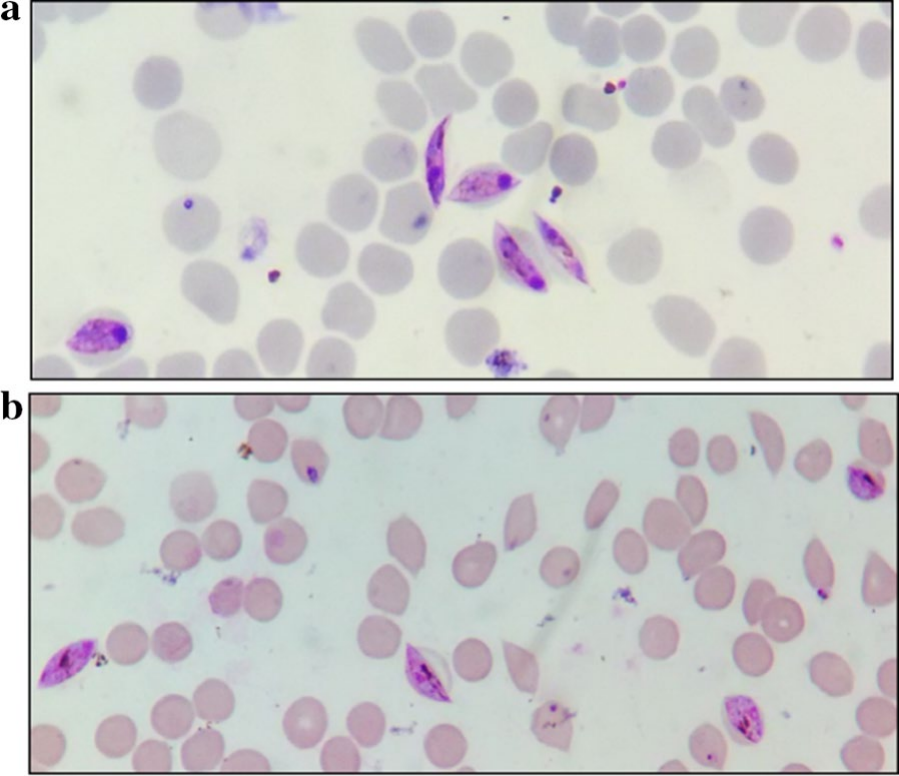
**a** Untreated *P. falciparum* gametocytes. **b** Primaquine treated gametocytes. Untreated gametocytes display normal morphology with intact chromatin and cytoplasm. Gametocytes treated with as high as 25 µM primaquine also display normal morphology without any morphological aberrations, hence demonstrating absence of any significant in vitro gametocytocidal effect of primaquine

Methylene blue was effective in targeting both early and late stage gametocytes produced from field isolates RKL-9 and JDP-8 in a dose dependent manner with a mean IC_50_ (NM) value of 424.1 nM (IC_50_ Total = 958.1 nM) for early stage and 106.4 nM (IC_50_ Total = 1060.2 nM) for late stage gametocytes. Individual IC_50_ value for each field isolate in ‘NM’ and ‘Total’ category is mentioned in Table 3. Concentration–response curves of early and late stage gametocytes for NM category (normal morphology, data include healthy gametocytes with no visible drug induced deformation) and Total (data include gametocytes with normal morphology and altered morphology, both) are shown in Fig. 2. Methylene blue was more effective in inducing morphological deformations in late stage gametocytes group as compared to early stage gametocyte group as evident from their IC_50_ values for NM category. These morphological abnormalities induced by methylene blue comprised of shrinkage and distortions that created degenerated or irregular gametocytes (Fig. 3). Moreover, many of the treated gametocytes appeared to have lost their outer membrane or developed membrane deformations. On the other hand, untreated gametocytes of the control group appeared perfectly healthy based on morphology and staining characteristics (Fig. 4a).

**Table 3:** Early and late stage IC_50_ values and log IC_50_ values along with 95% confidence intervals (CI) for methylene blue against gametocytes produced from RKL-9 and JDP-8

Isolate	Early stage gametocytes				Late stage gametocytes			
	NM		Total		NM		Total	
	IC_50_ (95% CI)	log IC_50_ (95% CI)	IC_50_ (95% CI)	log IC_50_ (95% CI)	IC_50_ (95% CI)	log IC_50_ (95% CI)	IC_50_ (95% CI)	log IC_50_ (95% CI)
RKL-9	378.5 (239.1–599)	2.578 (2.379–2.777)	1069 (139.7–8180)	3.029 (2.145–3.913)	128.6 (71.06–232.9)	2.109 (1.852–2.367)	1235 (621.4–2455)	3.092 (2.793–3.390)
JDP-8	475.2 (318.2–709.7)	2.677 (2.503–2.851)	858.8 (477.5–1545)	2.934 (2.679–3.189)	88.09 (3.349–2317)	1.945 (0.5250–3.365)	910.1 (653.2–1268)	2.959 (2.815–3.103)

Methylene blue demonstrated submicromolar IC_50_ values (NM) for both early and late stage gametocytes produced from RKL-9 and JDP-8 (All IC_50_ values are in nanomolar). No significant difference was observed between logarithm of IC_50_ values (NM and total) obtained for methylene blue against RKL-9 and JDP-8 (both early and late stage gametocytes) (p > 0.05)

Primaquine which was tested under similar conditions showed lack of any gametocytocidal activity even at micromolar concentrations. Healthy gametocytes (both early and late category) were observed in primaquine treated group even at concentrations as high as 25 µM (Fig. 4b). Here, gametocytes appeared perfectly non-compromised and looked similar to untreated control group (Fig. 4a).

## Discussion

This work highlights the applicability of culture adapted field isolates of *P. falciparum* in anti-gametocyte drug discovery. Here, we present a simple technique to produce gametocytes in high yield from gametocyte producing field isolates, useful for gametocytocidal drugs screening applications. To date, limited data is available involving field isolates for directly testing gametocytocidal activity. This might be because very few isolates can reproducibly generate gametocytes in high yield in vitro and also show a gradual loss of gametocyte producing potential in continuous culture [23]. This makes it difficult to study the process of gametocytogenesis [23, 24] and to perform gametocytocidal drug screening due to the dependency on cryopreserved stabilates with minimum passage in order to preserve gametocyte production phenotype [13]. The two field isolates, RKL-9 and JDP-8 used in present study did not show any significant reduction in gametocyte production potential in vitro for at least 6 months in asexual culture. This gives these isolates an additional advantage over clonal lines such as 3D7 in which ability to form gametocytes wanes in as little as 2 weeks [25]. Moreover, gametocyte production potential in RKL-9 and JDP-8 appeared to be stable after multiple freeze–thaw cycles. Other studies also reported no loss in gametocyte production upon maintenance of isolates in asexual culture for 18 months [22] and also after cryopreservation [13, 26, 27]. In vitro gametocyte production potential of parasite is strain specific [28], exhibited in response to ‘nonspecific’ stress in the form of environmental stimuli [22, 23, 29]. However, the definition of ‘stress’ as well as other triggers involved in pathway shift towards gametocytogenesis in *P. falciparum* are not precisely clear [30]. The stress on the parasite is not regulated by a single component but might be a collective contribution of multitude of factors, such as high parasite load, and decrease with haematocrit [23, 31]. Studies suggest that some clones show more preference towards production of gametocytes [26, 29, 32, 33] than others under similar conditions as a result of which gametocyte production in some isolates is upregulated [34, 35]. This is also evident from data reported here, as out of 15 culture adapted field isolates, only 2 (RKL-9 and JDP-8) were able to reproducibly produce > 2% gametocytes in vitro. Moreover, asexual stages cultivated from both RKL-9 and JDP-8 were found to be chloroquine resistant. Production of higher gametocytaemia in vitro (and in vivo) by drug resistant parasites may be correlated with tendency to spread the resistant mutation as a part of parasite’s survival strategy [34, 36]. However, current study was not designed in that context and separate studies involving more number of field isolates are needed to be carried out before a link between drug resistance and in vitro gametocytogenesis can be established.

Herein, gametocytocidal activity of methylene blue which is primarily used for treatment of methemoglobinemia is evaluated amongst Indian field isolates of *P. falciparum*. In present study as well as other in vitro studies carried out using standard laboratory strains, methylene blue was able to effectively target gametocytes, especially relatively less metabolizing stage V [8–13, 28]. However, IC_50_ values obtained for methylene blue were inconsistent across all these studies (varied from 29.5 nM in [10] to 490 nM in [8] and 106.44 nM (late stage mean IC_50_, present study)]. In spite of differences in drug efficacies in multiple studies (might be due to variation in culture parameters including length of drug exposure, type of screening assay, and difference in parasite strain used [28]), methylene blue was effective in killing gametocytes across all these studies. In the present work, morphological alterations induced by methylene blue are described. These alterations comprise of shrinkage, distortions and membrane deformations clearly representing unhealthy gametocytes. However, it is difficult to directly correlate morphology with viability. Therefore, viability and infectivity of these morphologically deformed gametocytes remains to be evaluated. Moreover, primaquine is a gametocytocidal drug having in vivo activity [5] but data reported here identifies it as non-gametocytocidal. This disparity between efficacy data highlights the absence of any metabolic activation in vitro because of lack of liver enzymes activity required for generation of active metabolites of primaquine [37]. However, identity of the metabolites and mode of action of primaquine is not fully elucidated [38]. Although, primaquine was not expected to show any significant potency in vitro but was added in present study to validate earlier studies [8, 9] and also served as an additional negative control for methylene blue other than DMSO. A major advantage that methylene blue confers over its alternatives is that, it is the only registered non 8-aminoquinoline having late stage gametocytocidal activity, which is inexpensive and currently, a suitable alternative to primaquine. So evaluation of gametocytocidal activity of methylene blue amongst Indian field isolates of *P. falciparum* has utmost importance. However, more evidence is needed to ascertain a dose that is safe for both G6PD deficient and G6PD non-deficient population as well as effective for stopping transmission. The study sets the stage for further basic and clinical research required for consideration of methylene blue as a gametocytocidal adjunct along with standard ACT in India and developing recommendations for future use.

## Conclusions

This is the first study as far as authors know which establishes culture adapted Indian field isolates as in vitro drug sensitivity model for screening gametocytocidal compounds. It is believed by the authors that field isolates should be utilized for validation of gametocytocidal hits (obtained by high throughput drug screening experiments using reference strains) and gametocyte producing field isolates described in this study such as RKL-9 and JDP-8 might play an important role in anti-gametocyte drug discovery. Methylene blue, which is currently, a suitable alternative to primaquine as a transmission blocking drug showed remarkable gametocytocidal effect in vitro, thereby inducing morphological deformations in treated gametocytes. This study highlights gametocytocidal properties of methylene blue amongst Indian field isolates and emphasizes the utility of field isolates in gametocytocidal drug screening. Transmission blocking potential of methylene blue should be further explored in ex vivo standard membrane feeding studies using Indian field isolates as well as clinical studies amongst Indian population. This will encourage future research that will help in forming recommendations for use of methylene blue as a transmission blocking drug in India.

## Abbreviations

G6PD: glucose-6-phosphate dehydrogenase
ACT: artemisinin combination therapy
NM: normal morphology
AM: altered morphology
